# Manual Acupuncture Regulates Behavior and Cerebral Blood Flow in the SAMP8 Mouse Model of Alzheimer’s Disease

**DOI:** 10.3389/fnins.2019.00037

**Published:** 2019-01-31

**Authors:** Ning Ding, Jing Jiang, Anping Xu, Yinshan Tang, Zhigang Li

**Affiliations:** ^1^School of Acupuncture-Moxibustion and Tuina, Beijing University of Chinese Medicine, Beijing, China; ^2^School of Nursing, Beijing University of Chinese Medicine, Beijing, China; ^3^Department of Rehabilitation in Traditional Chinese Medicine, The Second Affiliated Hospital of Zhejiang University School of Medicine, Hangzhou, China

**Keywords:** manual acupuncture, Alzheimer’s disease, behaviors, cerebral blood flow, MRI, hippocampus, prefrontal lobe, Morris water maze

## Abstract

**Background:** A growing body of evidence has demonstrated that cerebrovascular function abnormality plays a key role in occurrence and worsening of Alzheimer’s disease (AD). Reduction of cerebral blood flow (CBF) is a sensitive marker to early perfusion deficiencies in AD. As one of the most important therapies in complementary and alternative medicine, manual acupuncture (MA) has been used in the treatment of AD. However, the moderating effect of MA on CBF remains largely unknown.

**Objective:** To investigate the effect of MA on the behavior and CBF of SAMP8 mice.

**Methods:** SAMP8 mice were randomly divided into the AD, MA, and medicine (M) groups, with SAMR1 mice used as the normal control (N) group. Mice in the M group were treated with donepezil hydrochloride at 0.65 μg/g. In the MA group, MA was applied at Baihui (GV20) and Yintang (GV29) for 20 min. The above treatments were administered once a day for 15 consecutive days. The Morris water maze and arterial spin labeling MRI were used to assess spatial learning and memory in behavior and CBF respectively.

**Results:** Compared with the AD group, both MA and donepezil significantly decreased the escape latency (*p* < 0.01), while also elevating platform crossover number and the percentage of time and swimming distance in the platform quadrant (*p* < 0.01 or *p* < 0.05). The remarkable improvement in escape latency in the MA group appeared earlier than the M group, and no significant statistical significance was observed between the N and MA group with the exception of days 5 and 10. The CBF in the prefrontal lobe and hippocampus in the MA group was substantially higher than in the AD group (*p* < 0.05) with the exception of the right prefrontal lobe, with similar effects of donepezil.

**Conclusion:** Manual acupuncture can effectively improve the spatial learning, relearning and memory abilities of SAMP8 mice. The increase in CBF in the prefrontal lobe and hippocampus could be an important mechanism for the beneficial cognitive effects of MA in AD.

## Introduction

Alzheimer’s disease (AD) is a neurodegenerative disease of the central nervous system characterized by a progressive loss of memory and cognitive impairment with high prevalence, high morbidity rate, and substantial medical costs. The main clinical manifestations include memory disorders, aphasia, apraxia, agnosia, incapacity for discernment, and changes in personality and behavior. AD is the most common causes of dementia, accounting for 70% of patients with dementia (Mayeux and Stern, 2012; Reitz and Mayeux, 2014). Forty-seven million people worldwide were living with dementia in 2015, and this number will reach 74.7 million in 2030. The global costs of dementia have increased from US$ 604 billion in 2010 to US$ 818 billion in 2015, an increase of 35.4% (Prince et al., 2015). With aging populations, the increasing financial and social burdens of AD establish it as a critically urgent public health concern (Jones et al., 2017).

A growing body of evidence indicates that cerebrovascular function abnormality plays a key role in the prevalence and severity of AD, and is also considered as a primary pathological feature of AD (Mazza et al., 2011; Li et al., 2014; Lourenco et al., 2017). Neurotoxins and ischemia-hypoxia caused by blood–brain barrier (BBB) dysfunction and oligemia can directly induce neuronal injury and synaptic dysfunction and promote amyloid-β (Aβ) accumulation by elevating amyloid precursor protein (APP) expression and reducing Aβ clearance, resulting in a neuroinflammatory response and initiation of the neurodegenerative process, eventually leading to dementia (Zlokovic, 2011; Sagare et al., 2012; Nelson et al., 2016). In terms of cerebral hypo-perfusion, reduction of cerebral blood flow (CBF) is a sensitive marker for the early perfusion deficiencies of AD (Lacalle-Aurioles et al., 2014; Mattsson et al., 2014; Wierenga et al., 2014). The robust association between reduced CBF and cognitive impairment underscores its critical role in the diagnosis and monitoring of AD (Hays et al., 2016; Wood, 2016; Leeuwis et al., 2017). Improving cerebrovascular function has become an important treatment strategy in AD (Thomason et al., 2013; Acost et al., 2017).

There are currently no effective medical treatments for AD (Selkoe, 2012; Hickman et al., 2016). In the decade from 2002 to 2012, the failure rates in clinical trials of AD treatments approached 100% (Vos et al., 2013). Manual acupuncture (MA), which is one of the most important alternative complementary treatments for AD, has positive outcomes due to its economic efficiency, convenience, and low rate adverse effects (Zhou et al., 2015). Research has demonstrated that MA can ameliorate symptoms, and improve the quality of life (Zeng et al., 2013; Jia et al., 2017; Zhou et al., 2017). In addition, MA has established benefits in improving sleep quality of patients with AD (Kwok et al., 2013; Simoncini et al., 2015). Mechanistic studies of MA have demonstrated that MA can not only affect functional activities in AD related brain areas (Wang et al., 2012; Tan et al., 2017; Zheng et al., 2018) and the default mode network (Liang et al., 2014; Wang et al., 2014a), but can also increase glucose uptake in the hippocampus (Jiang et al., 2015; Cao et al., 2017). However, the effects of MA on CBF in AD are unclear. Considering that CBF plays a central role in the pathogenesis and development of AD, it is vital to uncover how MA can impact the regulation of CBF in AD model.

Although the effects of MA on rodent behavior in the Morris water maze have been initially explored and the benign effects of MA on spatial learning and memory has been confirmed (Li et al., 2012; Dong W. et al., 2015; Luo et al., 2017), the experimental designs of some prior studies possess significant confounds. While studies have applied hidden platform and probe trials, reversal and visible platform trials are rarely included (Li et al., 2014, 2016; Wang et al., 2014b; Zhang et al., 2017). It is not possible to effectively exclude potential differences in motivational, visual and motor abilities, which makes it difficult to support strong conclusions concerning the effectiveness of MA as a behavioral intervention and compare its effects with drugs commonly used to treat AD. This limits the ability of these studies to guide clinical practice and associated mechanistic research. Therefore, the moderating effect of MA on AD related behavior in mouse models of AD requires additional study.

The current study explores the effects of MA on learning, memory, and CBF with the Morris water maze and arterial spin labeling (ASL) MRI in senescence-accelerated prone mouse 8 (SAMP8) and senescence-accelerated resistant mouse 1 (SAMR1), aiming to assess the interventional efficacy of MA in AD and provide objective imaging evidence. This is the first time, to the best of our knowledge, that a study has focused on the CBF response after MA treatment in AD. Our data represent a significant contribution to the identification of the effectiveness of MA in improving cognitive ability and CBF in a mouse model of AD.

## Materials and Methods

### Experimental Animals

Since age-related impairment in learning is not apparent in female SAMP8 mice (Flood et al., 1995), male SAMP8 and SAMR1 mice strains were used. The mouse strains were purchased from the Zhi Shan (Beijing) Academy of Medical Science and tested by Chinese Academy of Medical Sciences [Animal Lot: SCXK(Jing)2014-0011]. Both types of mice weighed 30.0 ± 2.0 g and were 8 months old. The animals were housed in Experimental Animal Center of Beijing University of Chinese Medicine at a controlled temperature (24 ± 2°C) under a 12-h dark/light cycle, with sterile drinking water and a standard pellet diet available *ad libitum*. All mice were acclimatized to the environment for 7 days prior to experimentation. Efforts were made to minimize the number of animals used and the suffering of the experimental animals.

### Animal Grouping and Intervention

Thirty SAMP8 mice were divided into three groups (*n* = 10 per group): the AD group, the MA group, and the medicine (M) group. Ten SAMR1 mice were used as the normal control (N) group.

In the MA group, the mice were immobilized in mouse bags. MA on Baihui (GV20) and Yintang (GV29) was applied for 20 min, with transverse puncturing at a depth of 2–3 mm. The disposable sterile acupuncture needles (0.25 mm × 13 mm) (Beijing Zhongyan Taihe Medicine Company, Ltd.) were used. During the MA on Baihui (GV20) and Yintang (GV29), twirling manipulation was applied every 5 min and lasted 15 s each time. Each needle was rotated bidirectionally within 90° at a speed of 180°/s. For the M group, donepezil hydrochloride tablets (Eisai China, Inc., H20050978) were crushed and dissolved in distilled water and were delivered to mice by oral gavage at a dose of 0.65 μg/g (Geerts et al., 2005). The above treatments were administered once a day for 15 consecutive days, with no treatment of the N or AD groups. The mice in the N, AD, and M groups received the same 20 min restriction as the MA group. The duration of MA and selection of the acupoints was based on findings from our previous studies (Jiang et al., 2015, 2016, 2018; Cao et al., 2017; Ding et al., 2017). The above intervention lasted throughout the Morris water maze test period.

### The Morris Water Maze Test

At 24 h after the treatments for 15 consecutive days, mice in each group were used for the Morris water maze test. To assess learning and memory, the visible platform trial, hidden platform trial, probe trial and the reversal trial were conducted in order (Vorhees and Williams, 2006; Wu et al., 2016).

The Morris water maze consisted of a circular tank (diameter: 90 cm; height: 50 cm) filled with water to a depth of 30 cm, maintained at 24 ± 1°C, and rendered opaque with black ink. A removable circular platform (diameter: 9.5 cm; height: 28 cm) with the top surface 1 cm below the water was located inside the pool. The maze was designated by two principal axes, each line bisecting the maze perpendicular to one another. The end of each line demarcated four cardinal points which were used for four start locations: North (N), South (S), East (E), and West (W). The pool area was conceptually divided into four quadrants (NE, NW, SW, and SE) of equal size. Visual cues of different shapes were placed on the tank wall of each quadrant in plain sight of the mice. The experiment room was designed to maintain sound insulation, with an indirect light source and a low-light environment, and the remaining objects in this room were kept in their original locations. The experimental conditions were unchanged for the duration of the test. The data was automatically collected by a video camera (TOTA-450d, Japan) fixed to the ceiling and connected to a video recorder with an automated tracking system (China Daheng Group, China).

### Visible Platform Trial

The visible platform trial was used to exclude the influence of motivational or sensorimotor factors upon learning and memory performance on day 1. The platform was located 1 cm above the water surface in the middle of quadrant, and each mouse was released from one of four start locations and given 60 s to search for the visible platform. At the end of each trial, each mouse was placed on the platform or allowed to stay there for 10 s. Each animal was subjected to sessions of four trials. Each subsequent trial involved a different platform location and starting direction. The time to find the platform was recorded as escape latency, and the swimming speed was analyzed by EthoVision (3.1.16, Noldus).

### Hidden Platform Trial

The hidden platform trial was performed in order to assess learning from days 2 to 6. The platform was positioned in the middle of the SW quadrants. Mice were given a series of daily trials using a semi-random set of start locations. The four start locations were used with the restriction that one trial each day was from each of the four positions. Each mouse was released from one of four start locations and had 60 s to search for the hidden platform. At the end of each trial, the mouse was placed on the platform or allowed to stay there for 10 s, 4 trials per day were performed for 5 consecutive days, with the visual cues kept constant. The escape latency was recorded for subsequent analysis.

### Probe Trial

To assess reference memory, the probe trial was conducted on day 7. The platform was removed and each mouse was placed in the pool once for 60 s. The starting location was the farthest quadrant from the SW quadrant, the NE quadrant. The swimming distance in the maze was recorded, and the platform crossover number, swimming speed, and percentage of the swimming distance in the platform quadrant were analyzed.

### Reversal Trial

To evaluate the reversal learning ability, the reversal trial was performed from days 8 to 11. The platform was positioned in the middle of the NE quadrant, and the procedures for the reversal trial were the same as used in the hidden platform trial.

### Arterial Spin Labeling MRI

At 24 h after the Morris water maze, six mice in each group were randomly selected and used for the ASL MRI. The magnetic resonance tomograph “PharmaScan” US 70/16, 7.0 T, 300 MHz (Bruker, Ettlingen, Germany) was applied. During the MRI scanning, mice were anesthetized with isoflurane (3% for induction, 1–2% for maintenance) mixed with oxygen (1 L/min), delivered through a nasal mask. Respiration rate was monitored using an SA Instruments Model 1025 monitoring and gating system (Stony Brook, NY, United States) and maintained throughout the experimental period at 50–70 breaths per min by adjusting isoflurane levels. Body temperature was maintained at approximately 37°C using a circulating water system (SC100-S14P, Thermo Scientific).

The T2WI images were acquired with fast spin-echo pulse sequence, repetition time (TR) = 3930 ms, echo time (TE) = 35 ms, image size = 256 × 256, field of view (FOV) = 20 min × 20 mm. The CBF images were obtained from continuous ASL with echo-planar imaging fluid-attenuated inversion recovery (EPI-FLAIR) sequences. Acquisition parameters were TR/TE = 10000/79 ms, image size = 256 × 256, FOV = 20 mm × 20 mm. The CBF images were reconstructed with paravision version 5.1 software (Bruker, PharmaScan, Germany). The bilateral CBF of the prefrontal lobe and hippocampus were calculated and compared between groups.

### Statistical Analysis

The statistical analysis was performed using the SPSS software, version 17.0 (SPSS, Inc., Chicago, IL, United States), and the data were expressed as the mean ± standard deviation. Two-way ANOVA with repeated measures was used to analyze group differences in the escape latency. A one-way ANOVA followed by LSD multiple-range test was used to analyze group differences in the visible platform, probe trial and CBF. For the non-normally distributed data or for data with heterogeneous variance, a Kruskal–Wallis test was used. Statistical significance was set to *p* < 0.05 and high statistical significance was set to *p* < 0.01.

## Results

### Effect of MA on Spatial Learning

**FIGURE 1:**
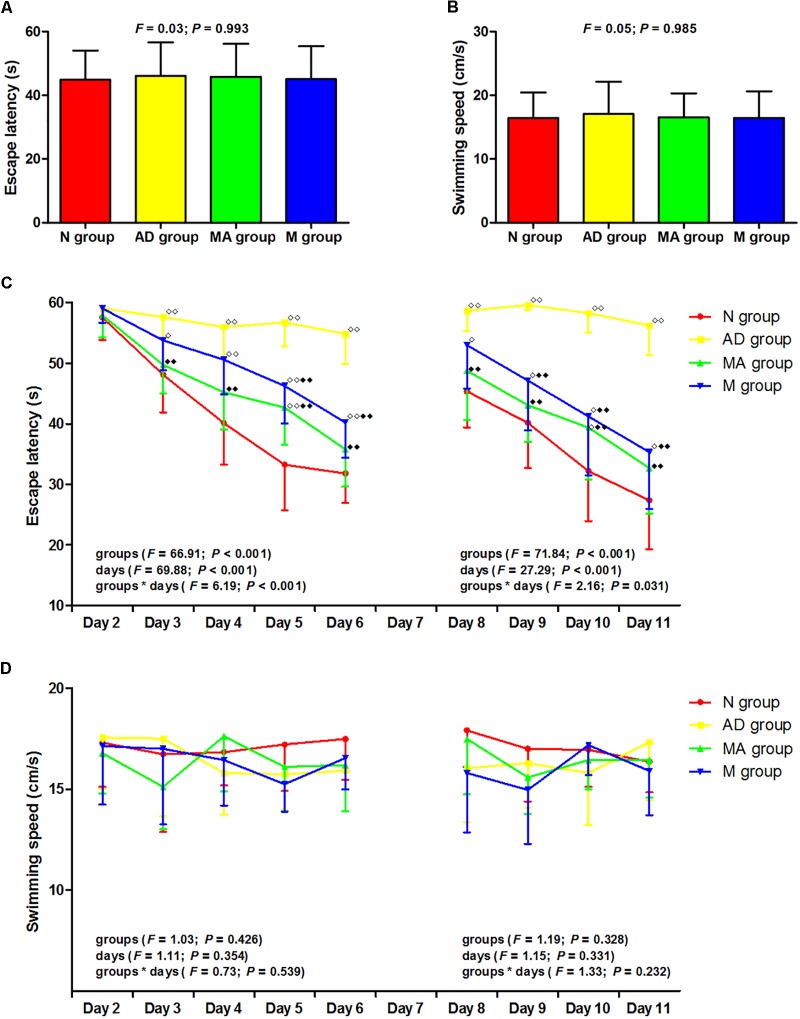
The results of the visible platform, hidden platform, and reversal trial in each group (*n* = 10, mean ± SD). **(A,B)** Comparison of the escape latency and swimming speed of all groups in the visible platform. One-way ANOVA was used. **(C)** Comparison of the escape latency of all groups in the hidden platform and reversal trial. Two-way ANOVA with repeated measures was used. LSD-t was presented in Supplementary Tables 1, 2. **(D)** Comparison of the swimming speed of all groups in the hidden platform and reversal trial. Two-way ANOVA with repeated measures was used. ^
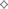

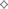
^*P* < 0.01, ^
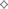
^*P* < 0.05 compared with the N group. ^
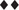
^*P* < 0.01 compared with the AD group.

The results of the visible platform, hidden platform, and reversal trial in the Morris water maze test are presented in Figure 1. In the visible platform trial, there were no significant differences in the escape latency or swimming speed among the groups. In the hidden platform and reversal trial, the escape latency of the N, MA, and M groups decreased gradually, but the AD group maintained a long latency. There were no significant group differences in the escape latency on day 2. Compared with the N group, the escape latency in the AD group significantly increased from days 3–6 to 8–11 (*p* < 0.01). The escape latency in the MA and M groups were notably lower than the N group on days 5 and 10 and from days 3–6 to 8–11, respectively (*p* < 0.01 or *p* < 0.05). Compared with the AD group, the escape latency in the MA and M groups were substantially decreased from days 3–6 to 8–11 and from days 5–6 to 9–11, respectively (*p* < 0.01). There were no significant group differences in the swimming speed in the hidden platform and reversal trial.

### Effect of MA on Spatial Memory

**FIGURE 2:**
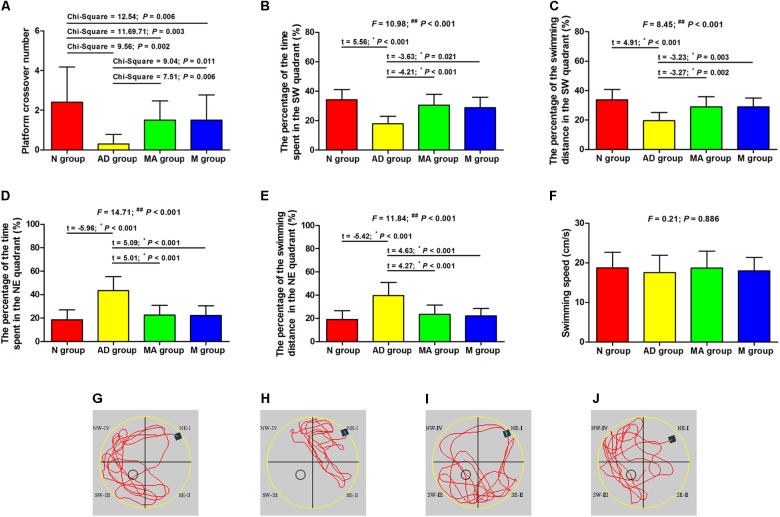
The results of the probe trial in each group (*n* = 10, mean ± SD). **(A)** Comparison of the platform crossover numbers of all groups. **(B,C)** Comparison of the percentage of time and swimming distances in the SW quadrant of all groups. **(D,E)** Comparison of the percentage of time and swimming distances in the NE quadrant of all groups. **(F)** Comparison of the swimming speed of all groups. **(G–J)** Swimming trajectories in the N, AD, MA and M groups, the water entry points were shown by black square. One-way ANOVA followed by LSD multiple-range test was used except in the comparison of the platform crossover numbers, which were analyzed by Kruskal–Wallis test. *^∗^p* represents the *post hoc* analysis. ^##^*p* < 0.01.

The results of the probe trial in the Morris water maze test are presented in Figure 2. The platform crossover number and the percentage of time and swimming distance in the SW quadrant in the AD group was significantly lower than in the N group (*p* < 0.01), and the percentage of time and swimming distance in the NE quadrant were significantly increased (*p* < 0.01). The platform crossover number, the percentage of time and swimming distance in the SW quadrant in the MA and M groups were higher compared to the AD group (*p* < 0.01 or *p* < 0.05), whereas the platform crossover frequency was still lower than the N group (*p* < 0.01). The percentage of time and swimming distance in the NE quadrant in the MA and M groups were significantly lower than the AD group (*p* < 0.01). No significant difference on the swimming speed among the groups was observed. As for search strategy, swimming activity was mostly concentrated in the SW quadrant in the N, MA, and M group. In contrast, the swimming activity of the AD group was mostly in the NE quadrant.

### The Effect of MA on CBF of Prefrontal Lobe and Hippocampus

**FIGURE 3:**
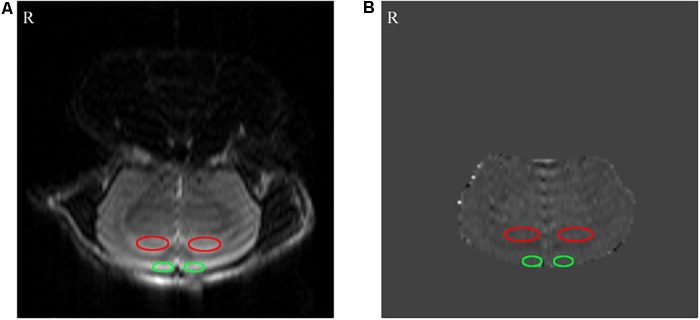
The results of the arterial spin labeling (ASL) MRI. **(A)** Perfusion FAIR-RARE images. **(B)** CBF images. The bilateral hippocampus and prefrontal lobe are shown by red and green ellipses, respectively.

The effect of MA on CBF in the prefrontal lobe and hippocampus are presented in Figure 3, 4. The bilateral CBF of the prefrontal lobe and hippocampus in the AD group was significantly lower compared to the N group (*p* < 0.01). Compared with the AD group, bilateral CBF of the prefrontal lobe and hippocampus was increased in the MA and M groups (*p* < 0.01 or *p* < 0.05), yet levels in these groups were still substantially lower compared to the N group (*p* < 0.01 or *p* < 0.05). There was no significant difference in the CBF of the right prefrontal lobe between the MA and AD groups.

**FIGURE 4:**
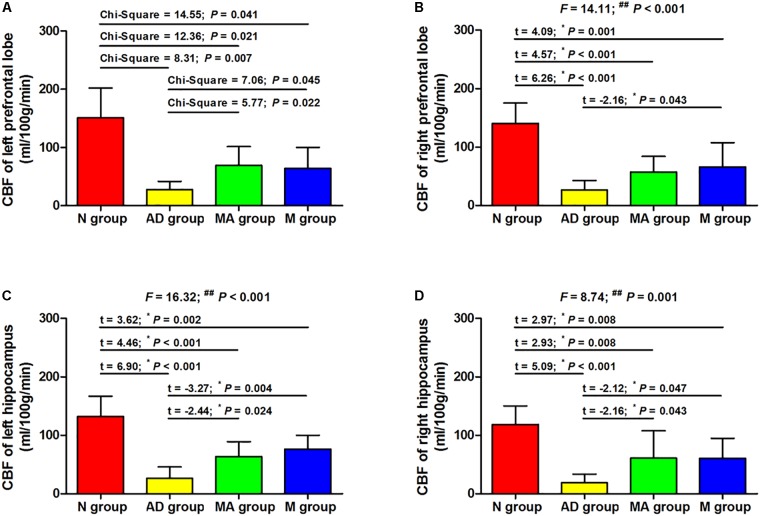
The comparison of the CBF of all groups (*n* = 10, mean ± SD). **(A,B)** The bilateral prefrontal lobe. **(C,D)** The bilateral hippocampus. One-way ANOVA followed by LSD multiple-range test was used except in the comparison of the left prefrontal lobe, which were analyzed by Kruskal–Wallis test. *^∗^p* represents the *post hoc* analysis. ^##^*p* < 0.01.

## Discussion

As the classic behavioral experiment and de-facto standard for testing hippocampal function in laboratory rodents, the Morris water maze can effectively and objectively evaluate learning and memory ability (Garthe and Kempermann, 2013). The Morris water maze has advantages such as minimal training, a lack of food deprivation, insensitivity to differences in body weight and appetite, and repeated testing ability (Vorhees and Williams, 2014), and has been widely used in behavior testing related to learning and memory in a broad range of neurobiology topics. The results of the visible platform in this study indicate an absence of differences in escape latency and swimming speed among groups, supporting the presence of the identical motivational, visual and motor abilities in the N, AD, MA, and M groups. Also, no significant group differences in swimming speeds in hidden platform, probe and reversal trial were noted. Potential behavioral confounds of differences in the motivational, visual and motor abilities of groups can be excluded.

The results of the hidden platform, probe trial and reversal trial tests suggest that the escape latency in the AD group was significantly longer than the N group from days 3 to 6 and from days 8 to 11 (*p* < 0.01), whereas the platform crossover number, percentage of time, and swimming distance in the SW quadrant were decreased (*p* < 0.01). The results indicate a remarkable decline in learning and memory ability in the AD group, in agreement with the typical pathological progression AD. It is worth noting that both percentage of time and swimming distance in the SW quadrant in the AD group were approximately 20%, which is consistent with a previous study (Wang et al., 2013). The reason for the variable distribution across the four quadrants was related to the preference of AD group, which was confirmed by the swimming activity in this group. The percentage of the time and swimming distance in the NE quadrant in the AD group were both higher compared to the N group (*p* < 0.01), demonstrating impaired learning and memory abilities in the AD group.

Both MA and donepezil significantly decreased the escape latency and increased platform crossover number and percentage of time and swimming distance in the SW quadrant (*p* < 0.01 or *p* < 0.05), indicating improvements in learning, memory and reversal learning ability with MA which were equal to donepezil. However, it should be noted that the specific effects of MA differed compared to donepezil. In the hidden platform and reversal trials, the significant decrease in escape latency in the MA group occurred from days 3–6 to 8–11 (*p* < 0.01). Similar changes were observed in the M group on days 5–6 to 9–11 (*p* < 0.01). Additionally, there were no significant differences in escape latency between the N and MA groups with the exception of days 5 and 10 (*p* < 0.01 or *p* < 0.05), and decreases in escape latency between the N and M groups were observed from days 3–6 to 8–11 (*p* < 0.01 or *p* < 0.05). These results suggest that MA tends to have a greater effect in improving spatial learning ability, and presents therapeutic advantages of high efficacy. Considering the other advantages of MA, including economic feasibility and low risk of adverse effects, additional studies of the effects of MA on AD should be encouraged.

The different pattern in improving spatial learning ability may be attributed to the characteristics of multiple targets of MA. The primary mechanism of donepezil is inhibiting acetylcholinesterase activity. In contrast, the effects of MA on AD involve multiple mechanisms, including anti-inflammatory (Ding et al., 2017), anti-apoptotic (Guo et al., 2015, 2016), anti-antioxidant stress (Liu et al., 2013; Sutalangka et al., 2013), and the regulation of Aβ production (Dong W.G. et al., 2015). The multiple targets of MA may explain the difference with donepezil in improving learning ability, and this characteristic addresses the need for multi-modal therapies for AD (Ubhi and Masliah, 2013). In general, the Morris water maze data in this study further confirmed the efficacy and reliability of MA in treating AD. The technical details, such as acupoints selection and manipulation and arrangement of treatment provide valid references for the clinical researchers.

The MRI results indicate that the CBF in prefrontal lobe and hippocampus in the AD group was significantly lower than the N group (*p* < 0.01), which parallel the pathological changes in CBF in AD. This study, for the first time to the best of our knowledge, confirmed that MA can increase CBF in the prefrontal lobe and hippocampus (*p* < 0.01 or *p* < 0.05). This effect of MA on CBF was equivalent to donepezil, and the effect of donepezil on CBF reported in this study is consistent with previous reports (Shimizu et al., 2015; Iizuka and Kameyama, 2017). The prefrontal lobe and hippocampus are necessary for spatial learning and memory, and the hippocampus is the primary substrate of spatial memory abilities and necessary for acquisition, retrieval and consolidation/storage of spatial information (D’Hooge and De Deyn, 2001; Curlik et al., 2014). The prefrontal-hippocampal circuit comprises the major navigation system in the rodent brain, where interconnections between the prefrontal cortex and dorsal striatum are more important for motivational or goal-directed aspects of spatial learning (Pooters et al., 2015). As we mentioned above, a decrease in CBF can directly cause ischemia and anoxia of the brain, resulting in neural injuries, neurological disorders, and initiating the neurodegenerative process. Therefore, we speculate that MA can protect against neuronal damage, delay the process of neurodegeneration, and maintain the structural and functional integrity of cognition related brain regions through enhanced CBF in the prefrontal lobe and hippocampus. Improving hemoperfusion of the brain could be one important mechanism for the effects of MA in AD.

As for potential mechanisms for how MA affects CBF, although the mechanism of decreased CBF has not been fully elucidated in AD, it was reported that structural and functional impairment of the cerebral microvasculature is involved (Dorr et al., 2012; Kimbrough et al., 2015). Therefore, the benign regulation of CBF by MA may be mediated through protective effects on cerebrovascular structure and function. Moreover, several studies have confirmed that Aβ deposition is a significant cause of cerebrovascular damage in AD (Hamel, 2015; Lai et al., 2015; Giannoni et al., 2016; Hecht et al., 2018). Our previous study also indicated that acupuncture can decrease the expression of Aβ and improve its clearance in the hippocampus and prefrontal lobe (Jiang et al., 2016; Wang et al., 2016). Therefore, we speculate that the benign regulation of CBF by MA was achieved via alleviation of the cerebrovascular impairment induced by Aβ. The effects of MA on Aβ-mediated impairment in cerebral microvasculature and CBF deserve further exploration.

It is worth noting that the CBF in the prefrontal lobes and hippocampus in the MA and M groups were still significantly lower than the N group (*p* < 0.01 or *p* < 0.05) despite the improvements compared to the AD group (*p* < 0.01 or *p* < 0.05). Researches showed that SAMP8 mice present age-related deterioration in behavior, physiology, neuropathology, and neurochemistry (Takeda, 2009). These mice exhibit Aβ deposition in the hippocampus as early as 6 months that progresses with age. In contrast, this process does not start in SAMR1 mice until 15 months (Del Valle et al., 2010). Thus, we speculate that the limited beneficial effects of MA and donepezil on CBF are due to the advanced age of SAMP8 mice in this study, suggesting that AD treatment should be initiated at an early stage.

This study has some limitations in the following areas. Firstly, there is a need for the optimization of MA in future studies, especially with respect to intervention time and duration. The limited effect of MA on CBF in this study suggests that early and longer interventions are necessary. In addition, a larger sample size and repeated behavioral measure before and after MA should be considered for future studies. Due to the different patterns of MA and donepezil in improving spatial learning ability in this study, more work needs to be devoted to confirming and clarifying the characteristics of the beneficial effects of MA in treating AD.

In summary, this study reported the beneficial effects of MA on spatial learning, reversal learning, and memory in SAMP8 mice. These effects of MA were equivalent to donepezil and present therapeutic advantages of high efficacy. We confirmed, for the first time to the best of our knowledge, that MA can effectively enhance CBF in the prefrontal lobes and hippocampus. The potential protective effects of MA on Aβ-mediated cerebrovascular impairment deserve further exploration.

## Ethics Statement

All experimental procedures complied with the guidelines of the “Principles of Laboratory Animal Care” formulated by the National Institute of Health and the legislation of the People’s Republic of China for the use and care of laboratory animals. The experimental protocols were approved by the Medicine and Animal Ethics Committee of the Beijing University of Chinese Medicine.

## Author Contributions

ND: experimental design, data analysis, and manuscript preparation. JJ: experimental design and manuscript preparation. AX and YT: data collection. ZL: experimental design. All authors contributed to draft the manuscript and have read and approved the final manuscript.

## Conflict of Interest Statement

The authors declare that the research was conducted in the absence of any commercial or financial relationships that could be construed as a potential conflict of interest.
