# Supplementary material for: Manual Acupuncture Regulates Behavior and Cerebral Blood Flow in the SAMP8 Mouse Model of Alzheimer’s Disease
Source: Front Neurosci. 2019 Jan 31;13:37. doi: 10.3389/fnins.2019.00037 (PMC6365452; doi:10.3389/fnins.2019.00037)
Supplement: Supplementary file 1 [file Table_1.DOC]

**SUPPLEMENTARY TABLE 1 | The LSD-t of escape latency in hidden platform trial (t, P).**

| **Groups** | **Day 3** | **Day 4** | **Day 5** | **Day 6** |
| --- | --- | --- | --- | --- |
| N | - | - | - | - |
| AD | (-4.31; < 0.001) | (-5.95; < 0.001) | (-8.62; < 0.001) | (-9.39; < 0.001) |
| MA | (3.57; 0.001) | (4.05; < 0.001) | 1. MA (-3.46; 0.001)   AD-MA (5.16; < 0.001) | (7.75; < 0.001) |
| M | (-2.56; 0.015) | (-3.93; < 0.001) | N-M (-4.77; < 0.001)  AD-M (3.84; < 0.001) | N-M (-3.42, 0.002)  AD-M (5.95; < 0.001) |

**SUPPLEMENTARY TABLE 2 | The LSD-t of escape latency in reversal trial (t, P).**

| **Groups** | **Day 8** | **Day 9** | **Day 10** | **Day 11** |
| --- | --- | --- | --- | --- |
| N | - | - | - | - |
| AD | (-4.63; < 0.001) | (-6.85; < 0.001) | (-7.47; < 0.001) | (-8.45; < 0.001) |
| MA | (3.45; 0.001) | (5.82; < 0.001) | N-MA (-2.05; 0.047)  AD-MA (5.42; < 0.001) | (6.91; < 0.001) |
| M | (-2.66; 0.012) | N-M (-2.48; 0.018)  AD-M (4.38; < 0.001) | N-M (-2.59; 0.014)  AD-M (4.88; < 0.001) | N-M (-2.32; 0.026)  AD-M (6.12; < 0.001) |
